# Lobetyolin induces apoptosis of colon cancer cells by inhibiting glutamine metabolism

**DOI:** 10.1111/jcmm.15009

**Published:** 2020-01-28

**Authors:** Wei He, Weiwei Tao, Feng Zhang, Qian Jie, Yun He, Wei Zhu, Jiani Tan, Weixing Shen, Liu Li, Ye Yang, Haibo Cheng, Dongdong Sun

**Affiliations:** ^1^ Changshu Hospital Affiliated to Nanjing University of Chinese Medicine Changshu China; ^2^ School of Integrated Chinese and Western Medicine Nanjing University of Chinese Medicine Nanjing China; ^3^ School of Pharmacy Nanjing University of Chinese Medicine Nanjing China; ^4^ Collaborative Innovation Center of Jiangsu Province of Cancer Prevention and Treatment of Chinese Medicine Key Laboratory of Famous Doctors' Proved Recipe Evaluation and Transformation Under State Administration of Traditional Chinese Medicine Nanjing University of Chinese Medicine Nanjing China

**Keywords:** ASCT2, HCT‐116, Lobetyolin, p53

## Abstract

The purpose of the present study was to evaluate the anti‐cancer property of Lobetyolin on colorectal cancer and explore its potential mechanism. Lobetyolin was incubated with HCT‐116 cells in the absence or presence of ASCT2 inhibitor Benser or p53 inhibitor Pifithrin‐α. The levels of glutamine, glutamic acid, α‐ketoglutarate, ATP and GSH were determined to measure the glutamine metabolism. Annexin V‐FITC/PI staining and TUNEL assay were applied to estimate the apoptotic condition. The levels of ASCT2 were examined by RT‐qPCR, Western blot and immunofluorescence staining. The expressions of cleaved‐caspase‐3, caspase‐3, cleaved‐caspase‐7, caspase‐7, cleaved‐PARP, PARP, p53, p21, bax and survivin were detected using Western blot analysis. As a result, the treatment with Lobetyolin effectively induced apoptosis and glutamine metabolism in HCT‐116 cells through ASCT2 signalling. The inhibition of ASCT2 reduced the glutamine‐related biomarkers and augmented the apoptotic process. We further found that the effect of Lobetyolin on HCT‐116 was related to the expressions of p21 and bax, and transportation of p53 to nucleus. The inhibition of p53 by Pifithrin‐α promoted the inhibitory effect of Lobetyolin on ASCT2‐mediated apoptosis. Lobetyolin also exerted anti‐cancer property in nude mice. In conclusion, the present work suggested that Lobetyolin could induce the apoptosis via the inhibition of ASCT2‐mediated glutamine metabolism, which was possibly governed by p53.

## 1 INTRODUCTION

As one of the most common malignant cancer types, colon cancer remains the second leading cause of cancer‐related deaths worldwide. Colorectal tumour is highly ranked in the morbidity and even accounts for the 8.5% mortality. Colon cancer deeply influences the life quality and causes heavy economic burden on the patients and society.^1^ During the past few decades, great advance has been achieved on the exploration of therapeutic target and compound. Unfortunately, most drugs are not satisfied due to their resistance, side effect and limited efficacy in clinical. Thus, it is urgent to develop the new candidate compound for the intervention of colorectal carcinogenesis.

The cell metabolism which provides energy and substance for the proliferation of tumour has been recognized as the key feature of carcinogenesis. Growing evidence elicited that the augmented requirement of carbon and nitrogen source conduced to the reliance of cancer cell on glutamine. Glutamine serves as a crucial metabolic substrate in cancer cell and participates in tumour development and proliferation. Alanine‐serine‐cysteine transporter 2 (ASCT2), the membrane‐associated protein, is a broad specificity bidirectional transport of glutamine.^2^ As the mediator of glutamine uptake and metabolism, ASCT2 is involved in the aetiology of multiple cancers, such as breast cancer, prostate cancer and melanoma. It was reported that the inhibition of ASCT2 repressed reduced cancer cell growth and proliferation in LS174T cell line.^3^ Thus, it was assumed that ASCT2 might be a candidate target for the intervention of colon cancer, whereas the underlying role of ASCT2 in the pathogenesis of colorectal cancer remains not fully understood.

*Codonopsis pilosula* (Franch.) Nannf., the famous traditional Chinese medicine, has been used to enhance the immune system, suppress blood pressure, attenuate gastrointestinal function, improve appetite and treat gastric ulcer.^4^ Its bioactive compound, Lobetyolin, is the critical ingredient of polyacetylenes in *C pilosula*. Growing evidence indicated that the Lobetyolin exerted anti‐inflammatory, anti‐oxidative^5^, ^6^ and xanthine oxidase inhibiting properties.^7^ It was noteworthy that Chinese medicine Bu‐Fei decoction, containing the constituent of Lobetyolin, was proved to relieve epithelial‐mesenchymal transition of non–small‐cell lung cancer.^8^ Moreover, the steamed *Codonopsis lanceolate*, which consisted of Lobetyolin, was also confirmed to show beneficial effect on H22 tumour‐bearing mice.^9^ Nevertheless, there has been limited report focused on the anti‐cancer effect of Lobetyolin in colon cancer. Thus, the present study was carried out to investigate the pharmacological effect of Lobetyolin on colon cancer and explore its underlying mechanism.

## 2 MATERIALS AND METHODS

### 2.1 Reagents

The tested compounds were provided by Dongdong Sun (Nanjing University of Chinese Medicine). For the treatment, the compounds were dissolved in culture medium and DMSO [with DMSO less than 0.1%(v/v)]. HCT116 colorectal cancer cells were supplied from the cell bank of the Chinese Academy of Sciences (Shanghai, China). RPMI 1640 culture medium was provided by Biological Industries (Beit‐Haemek). TUNEL apoptotic commercial kit was produced by Beyotime. Annexin V‐PI kit was purchased from KeyGEN. α‐ketoglutarate as obtained from Jinglai Biotechnology. The commercial kits for ATP, glutamine, glutamate and glutathione were provided by Jiancheng Biotechnology. The ASCT2 inhibitor GPNA was produced by Sigma‐Aldrich. Pifithrin‐α, the selective inhibitor for P53, was obtained from APExBIO. ASCT2 (#ab84903, 1:1000), P53 (#ab26, 1:1000), caspase‐3 (#ab197202, 1:1000), caspase‐7 (#ab69540, 1:1000), cleaved‐caspase‐3 (#ab2302, 1:1000), cleaved‐caspase‐7 (#ab2323, 1:1000), cleaved‐PARP (#ab32064, 1:1000), PARP (ab74290, 1:1000) and survivin (ab76424) were purchased from Abcam.

### 2.2 Cell culture

The HCT‐116 and NCM460 cells were cultured in RPMI 1640 medium complemented with 10% foetal bovine serum (Thermo) and 1% penicillin‐streptomycin (Biological Industries) at 37°C with 5% CO_2_ in a humidified incubator. The culture medium was refreshed every 1‐2 days.

### 2.3 MTT assay

1 × 10^4^/mL HCT‐116 cells were seeded on each well of 96‐well plate for 24 hours. The culture medium was refreshed, and the cells were treated with various concentrations of the compounds (1, 5, 10, 20, 40, 80 μmol/L) for 24 hours. Then, the cells were incubated with 20 μL 5 mg/mL 3‐(4,5‐Dimethyl‐2‐thiazolyl)‐2,5‐diphenyl‐2H‐tetrazolium bromide (MTT) for another 4 hours. Thereafter, the medium was discarded and 150 μL formazan crystals were dissolved in DMSO. The optical density was measured using a microplate spectrophotometer (Tecan) at 570 nm. The inhibition ratio was calculated according to the following formula: Cell viability (%) = (*A*_Treated_/*A*_Control_) × 100%.

### 2.4 The determination of glutamine, glutamic acid, α‐ketoglutarate, ATP and GSH

The concentrations of glutamine, glutamic acid, α‐ketoglutarate, ATP and GSH were measured in accordance with the procedure of manufacturer's instructions.

### 2.5 Annexin V‐FITC/PI staining

The apoptosis was evaluated by Annexin V‐FITC/PE staining. HCT‐116 cells were washed using cold PBS for two times and resuspended in 500 μL binding buffer. Afterwards, the cells were exposed to 1 μL V‐FITC/PE solution in dark environment at 25°C for 10 minutes. The percentage of apoptotic cell was analysed in a FACS flow cytometer (Becton Dickinson).

### 2.6 TUNEL assay

The apoptotic situation of HCT‐116 cells was estimated with terminal deoxynucleotidyl transferase‐mediated dUTP nick‐end labelling (TUNEL) assay. The cells were seeded on the chamber slides and treated with Lobetyolin for 24 hours. After washing with PBS, the TUNEL staining was carried out in accordance with the manufacturer's procedure. Anti‐evaporation membranes were applied to maintain the humidification. The anti‐fluorescence quenching solution was used prior to the visualization under fluorescence microscope. The experiment was conducted in triplicate.

### 2.7 Animals

6‐week‐old athymic nude (nu/nu) mice (SPF grade), obtained from Nanjing University of Chinese Medicine (Nanjing, China), were acclimatized in standardized environment (23 ± 2°C, 60 ± 10% humidity, 12‐hour light/dark cycle). The experimental procedures were approved and performed according to the guidelines set out by the Institutional Animal Care and Use Committee of Nanjing University of Chinese Medicine.

The mice were subcutaneously inoculated with HCT116 cells (2 × 10^6^ cells) at the right flank. 24 hours after tumour transplantation, the mice were randomly assigned into 4 groups (n = 10): control group, Lobetyolin (10 mg/kg), Lobetyolin (20 mg/kg) and Lobetyolin (40 mg/kg). The mice were intraperitoneally injected with Lobetyolin, and the control group intraperitoneally received vehicle for 2 weeks. Afterwards, the mice were killed and the tumours were removed and weighed. The tumours were applied for mRNA and Western blot analyses.

### 2.8 RT‐qPCR

2 mL 2.5 × 10^5^/mL HCT‐116 cells were seeded on the six‐well plate for 24 hours. Then, the culture medium was removed and the compound was added to the cells. 24 hours later, the cells were harvested for the RT‐qPCR assay. Total cellular RNA was isolated using TRIzol reagent. 1 μg total RNA was reverse transcribed to make cDNA using SYBR Premix Ex Taq™. The reaction mixer consisted of supermix, total RNA and DNase‐free water. RNA was transcribed using M‐MuLV Reverse Transcriptase (Invitrogen). The primers were Synthesized by Sangon Biotech, and the sequences were listed as Table 1.

**Table 1:** Primer sequences

Gene	Primer sequence (5′‐3′)
ASCT2	CCGCTTCTTCAACTCCTTCAA
	TGGATCATGTGGTACGCCCCTGT
BAX	CGAGCTGATCAGAACCATCA
	GGGGTCCCGAAGTAGGAA
Bcl‐2	GGAGGATTGTGGCCTTCTTTG
	GCATCCCAGCCTCCGTTATC
P21	CCT GTC ACT GTC TTG TAC CCT
	GCG TTT GGA GTG GTA GAA ATC T

### 2.9 Western blot

2 mL 2.5 × 10^5^/mL HCT‐116 cells were seeded on the six‐well plate for 24 hours. Then, the culture medium was removed and the compound 20 was added to the cells. 24 hours later, the cells were collected for Western blot analysis. The cells were washed twice with cold PBS, immersed in RIPA lysis buffer and centrifugated at 10 000 *g* for 10 minutes at 4°C. The protein concentration of the cells was determined using BCA commercial kit. Equal amount of sample was separated by 8%‐12% SDS‐polyacrylamide gel electrophoresis (SDS‐PAGE) and transferred onto PVDF membrane. The blots were blocked with 5% non‐fat milk and incubated with the corresponding primary antibodies at 4°C overnight. Afterwards, the cells were washed and incubated with secondary antibody conjugated with horseradish peroxidase for 1 hour. The membranes were incubated with ECL detective system and visualized. The relative intensities of protein bands were quantified by the Quantity one software.

### 2.10 Immunofluorescence staining

1 mL 5 × 10^5^ cells were seeded on six‐well plate for 24 hours. The culture medium was abandoned, and 2 mL new medium containing compound 20 was added to the cells. 24 hours later, the cells were fixed with 4% polyformaldehyde for 30 minutes. 150 μL first antibodies dissolved in BSA was incubated with the cells overnight at 4°C. The stained cells were then treated with goat anti‐rabbit Alexa Fluor in the dark for 1 hour and exposed to 150 μL DAPI. After sealing with anti‐fluorescence quenching solution, the samples were observed by fluorescence microscope.

### 2.11 Statistical analysis

All the results were illustrated in mean ± SD. One‐way analysis of variance with Student's *t* test was carried out using GraphPad 6.0. *P* < .05 was considered as significant.

## 3 RESULTS

### 3.1 The effect of Lobetyolin on HCT‐116 cells

**Figure 1:**
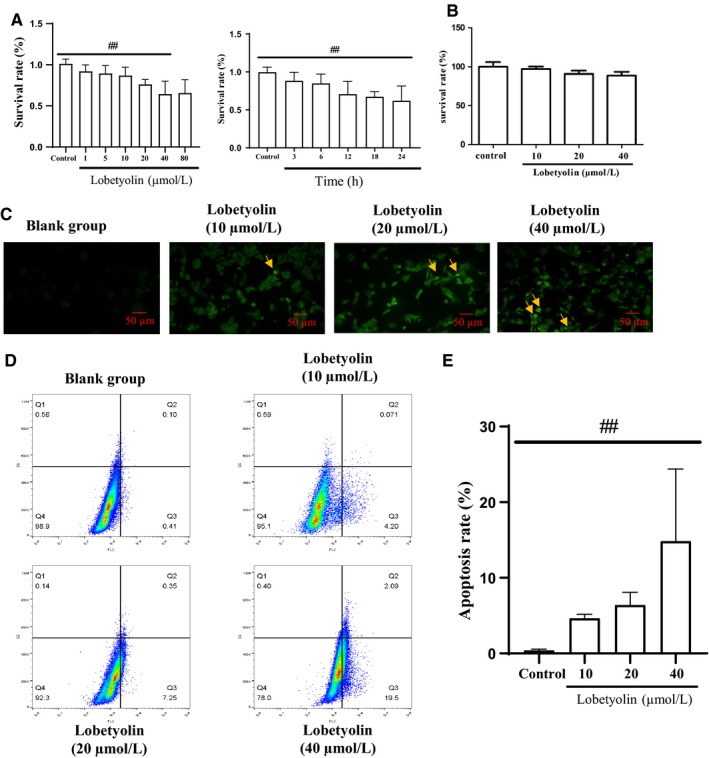
The effect of Lobetyolin on HCT‐116 cells. (A) The cell viability of Lobetyolin at different concentration and different time in HCT‐116 cells was measured by MTT assay; (B) The cell viability of Lobetyolin at different concentration in NCM460 cells was measured by MTT assay. (C) The effect of Lobetyolin on the apoptosis by TUNEL staining; (D) The effect of Lobetyolin on the apoptosis by Annexin V‐FITC/PI staining; (E) The apoptosis rate of Annexin V‐FITC/PI staining results. ##*P* < .01, compared with the control group

To identify the appropriate incubation time for Lobetyolin, HCT‐116 cells were treated with Lobetyolin for 3 hours, 6 hours, 12 hours, 18 hours and 24 hours. As shown in Figure 1A, The results of MTT assay indicated that Lobetyolin presented significant suppressing property at 10, 20 and 40 μmol/L in a concentration‐dependent manner (*P* < .01). Whereas, as there exhibits no obvious difference between 80 μmol/L and 40 μmol/L, we chose 10, 20 and 40 μmol/L for the further detection. Lobetyolin inhibited cell proliferation at 3 hours, 6 hours, 12 hours, 18 hours and 24 hours. Lobetyolin induced largest inhibitory effect on survival rate at 24 hours. In order to evaluate the cytotoxicity of Lobetyolin on normal cell, we performed the MTT assay in NCM460 cell. The data demonstrated that 10 μmol/L, 20 μmol/L and 40 μmol/L Lobetyolin did not significantly influence the cell viability in NCM460 cell (Figure 1B).

TUNEL staining was carried out to estimate the effect of Lobetyolin on the apoptosis. TUNEL‐positive cells, the key index for estimating apoptotic condition, were presented in Figure 1C. The treatment with 20 and 40 μmol/L Lobetyolin notably increased the number of positive cells compared with that of the control group. The Annexin V‐FITC/PI staining proved that the exposure to Lobetyolin (10, 20, 40 μmol/L) remarkably increased the population of early apoptosis, late apoptosis and necrosis compared with those of control group in concentration‐dependent manners. Lobetyolin (10, 20, 40 μmol/L) treatments prominently elevated the apoptosis rate compared with that in control group (*P* < .01). The above evidence depicted that the treatment with Lobetyolin (10, 20, 40 μmol/L) for 24 hours contributed to the inhibitory effect on cell viability and apoptosis in HCT‐116 cells (Figure 1D,E).

**Figure 2:**
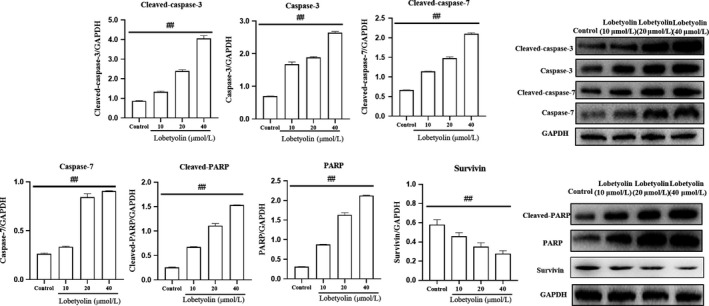
The effect of Lobetyolin on apoptosis‐related protein expressions in HCT‐116 cells. The effect of Lobetyolin on the expressions of cleaved‐caspase‐3, caspase‐3, cleaved‐caspase‐7, caspase‐7, cleaved‐PARP, PARP and survivin proteins in HCT‐116 cells by Western blot analysis; ##*P* < .01, compared with the control group

To examine the mechanism of Lobetyolin on apoptotic process, the protein expressions of cleaved‐caspase‐3, caspase‐3, cleaved‐caspase‐7, caspase‐7, cleaved‐PARP and PARP were examined. As illustrated in Figure 2, the protein levels of cleaved‐caspase‐3 and caspase‐3 were dramatically elevated with the incubation of Lobetyolin (10, 20, 40 μmol/L) compared with control group (*P* < .01). The treatment with Lobetyolin also effectively promoted the expressions of cleaved‐caspase‐7, caspase‐7 and *P* < .01. It was noteworthy that Lobetyolin (10, 20, 40 μmol/L) down‐regulated the expression of survivin. As expected, the generations of cleaved‐PARP and PARP were also augmented with the administrations of Lobetyolin (10, 20, 40 μmol/L, all *P* < .01).

### 3.2 The effect of Lobetyolin on the contents of Glutamine, glutamic acid, α‐ketoglutarate, ATP and GSH

**Figure 3:**
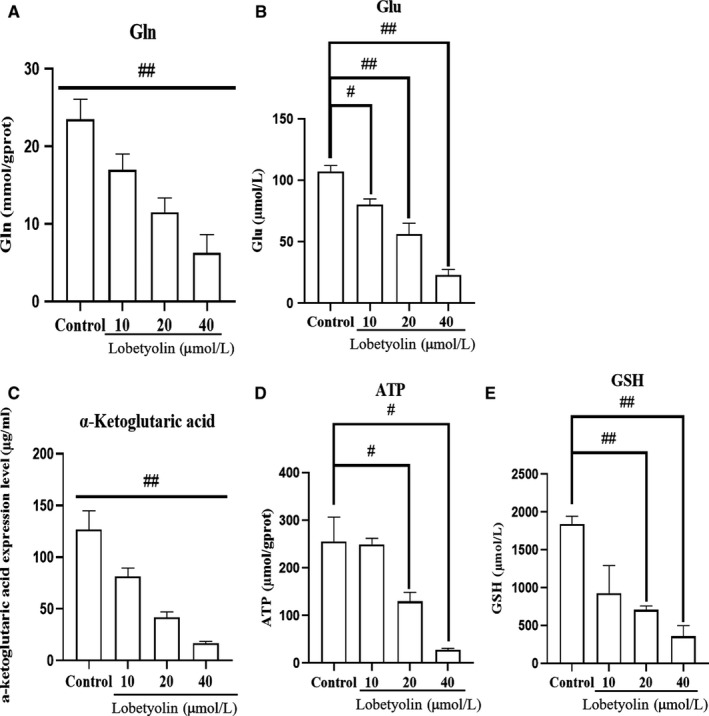
The effect of Lobetyolin on the contents of Glutamine, glutamic acid, α‐ketoglutarate, ATP and GSH. (A) The effect of Lobetyolin on the contents of Glutamine; (B) The effect of Lobetyolin on the contents of glutamic acid; (C) The effect of Lobetyolin on the contents of α‐ketoglutarate; (D) The effect of Lobetyolin on the contents of ATP; (E) The effect of Lobetyolin on the contents of GSH; #*P* < .05, ##*P* < .01, compared with the control group; Gln, Glutamine; Glu, glutamic acid; α‐ketoglutaric acid, α‐ketoglutarate

As illustrated in Figure 3, the treatment with Lobetyolin (10, 20, 40 μmol/L) evidently reduced the concentrations of glutamine and α‐ketoglutarate (all *P* < .01) compared with those in control group. Besides, the administration of Lobetyolin (20, 40 μmol/L) remarkably decreased the glutamic acid concentration (*P* < .01), which was more efficient than Lobetyolin (10 μmol/L, *P* < .05). Lobetyolin (20, 40 μmol/L) could also suppress the generation of ATP (*P* < .05) and GSH (*P* < .01), respectively. The analytical results suggested that Lobetyolin was capable of mediating glutamine metabolism.

### 3.3 The effect of Lobetyolin on ASCT2 and apoptosis

**Figure 4:**
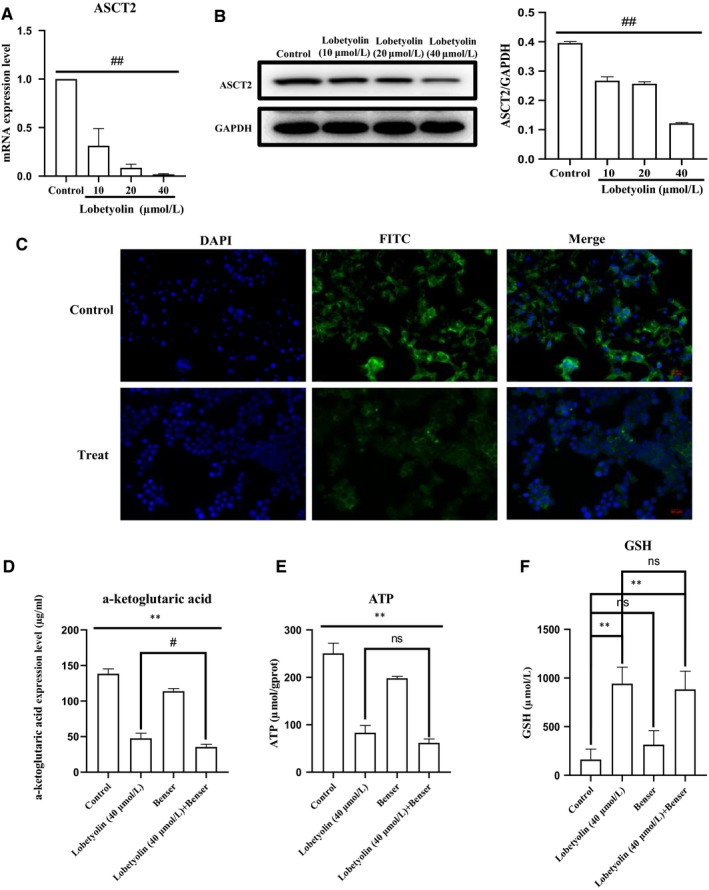
The effects of Lobetyolin on ASCT2 and apoptosis. (A) The effect of Lobetyolin on ASCT2 mRNA level in HCT‐116 cells; (B) The effect of Lobetyolin on ASCT2 protein expression in HCT‐116 cells by Western blot analysis; (C) The effect of Lobetyolin on ASCT2 expression levels in HCT‐116 cells by immunofluorescence assay; (D) The effect of Lobetyolin, Benser and Lobetyolin + Benser on the contents of α‐ketoglutarate; (E) The effect of Lobetyolin, Benser and Lobetyolin + Benser on the content of ATP; (F) The effect of Lobetyolin, Benser and Lobetyolin + Benser on the content of GSH; (G) The effect of Lobetyolin on cleaved‐caspase‐3, caspase‐3, cleaved‐caspase‐7, caspase‐7, cleaved‐PARP and PARP proteins in HCT‐116 cells; #*P* < .05, ##*P* < .01, compared with the control group; ##*P* < .01, compared with the Lobetyolin group

As depicted in Figure 4, the exposure to Lobetyolin (10, 20, 40 μmol/L) prominently reduced the ASCT2 mRNA levels (Figure 4A) and suppressed the ASCT2 protein expression (Figure 4B). Besides, the immunofluorescence assay also proved that Lobetyolin (40 μmol/L) treatment inhibited the expression of ASCT2 in HCT‐116 cells (Figure 4C).

Next, the HCT‐116 cells were stimulated with Benser, the selective inhibitor of ASCT2. The data demonstrated that the treatment with Lobetyolin (40 μmol/L), Benser and Lobetyolin (40 μmol/L) + Benser pronouncedly reduced the content of α‐ketoglutarate (Figure 4D, all *P* < .01). The Lobetyolin (40 μmol/L) and Lobetyolin (40 μmol/L) + Benser group significantly decreased the ATP concentration (*P* < .01) compared with that in control group, which were slightly more efficient than Benser group (*P* < .05) (Figure 4E). The administrations of the Lobetyolin (40 μmol/L) and Lobetyolin (40 μmol/L) + Benser also reduced the GSH level compared with that of control cells (Figure 4F, *P* < .05).

**Figure 5:**
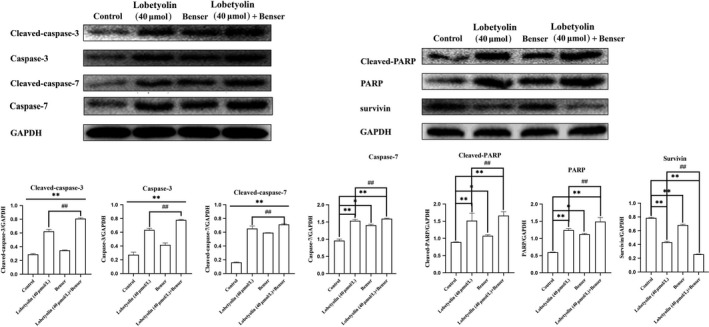
The effects of Lobetyolin (40 μmol/L), Benser and Lobetyolin (40 μmol/L) + Benser on apoptosis‐related protein expressions. The effect of Lobetyolin (40 μmol/L), Benser and Lobetyolin (40 μmol/L) + Benser on the expressions of cleaved‐caspase‐3, caspase‐3, cleaved‐caspase‐7, caspase‐7, cleaved‐PARP, PARP and survivin proteins in HCT‐116 cells; *P < .05, **P < .01, compared with the control group; ##*P* < .01, compared with the Lobetyolin group

In addition, the treatments with Lobetyolin (40 μmol/L), Benser and Lobetyolin (40 μmol/L) + Benser effectively inhibited the expressions of cleaved‐caspase‐3, caspase‐3 and cleaved‐caspase‐7 compared with control cells (all *P* < .01). The treatments with Lobetyolin (40 μmol/L) and Lobetyolin (40 μmol/L) + Benser notably down‐regulated the protein expressions of caspase‐7, cleaved‐PARP and PARP (*P* < .01), which was more potent than Benser group (*P* < .05). The administration of Lobetyolin (40 μmol/L), Benser and Lobetyolin (40 μmol/L) + Benser effectively promoted the expression of survivin (*P* < .01). Of note, Lobetyolin (40 μmol/L) + Benser group augmented the survivin expression more potential than that of Lobetyolin (40 μmol/L) group. The above evidence indicated the critical role of ASCT2 in the Lobetyolin‐mediated apoptosis in HCT‐116 cells (Figure 5).

### 3.4 The effect of Lobetyolin on the transportation of p53 to nucleus and the expressions of p21 and bax

**Figure 6:**
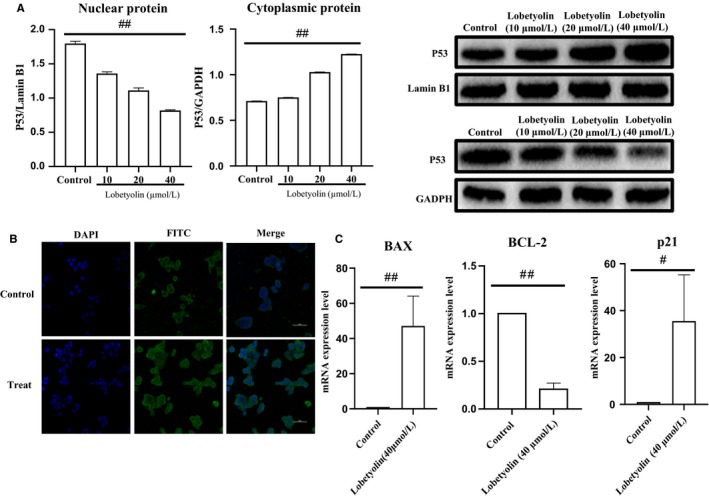
The effect of Lobetyolin on the transportation of p53 to nucleus, and the expressions of p21, Bcl‐2 and bax. (A) The effect of Lobetyolin on p53 protein transportation to the nucleus; (B) The effect of Lobetyolin on the p53 nucleoprotein expression by immunofluorescence assay; (C) The effect of Lobetyolin on the mRNA of apoptosis‐related BAX, Bcl‐2 protein and cell cycle–related p21 protein; #*P* < .05, ##*P* < .01, compared with the control group

Next, we investigated the effect of Lobetyolin on p53 protein transportation to the nucleus. As revealed in Figure 6A, the expression of p53 were down‐regulated in nucleus and up‐regulated in cytoplasmic (both *P* < .01) caused by the treatment with Lobetyolin (10, 20, 40 μmol/L). Figure 6B also revealed that the nucleoprotein expression of p53 was improved compared with that of control group. The obtained data confirmed that Lobetyolin administration promoted the translocation of p53 from cytoplasm to nucleus.

Moreover, the mRNA of apoptosis‐related protein p53 and cell cycle–related protein p21 were detected by RT‐qPCR. As shown in Figure 6C, the augmented mRNA levels of Bax (*P* < .01) and p21 (*P* < .05) were observed owing to the stimulation with Lobetyolin (40 μmol/L), while Bcl‐2 mRNA expression was significantly inhibited as result of the Lobetyolin (40 μmol/L) treatment. The data further displayed that the treatment with Lobetyolin could mediate the translocation of p53 to nucleus and attenuated the expressions of p21, Bax and Bcl‐2 in HCT‐116 cells.

### 3.5 The role of p53 in Lobetyolin‐mediated apoptosis

**Figure 7:**
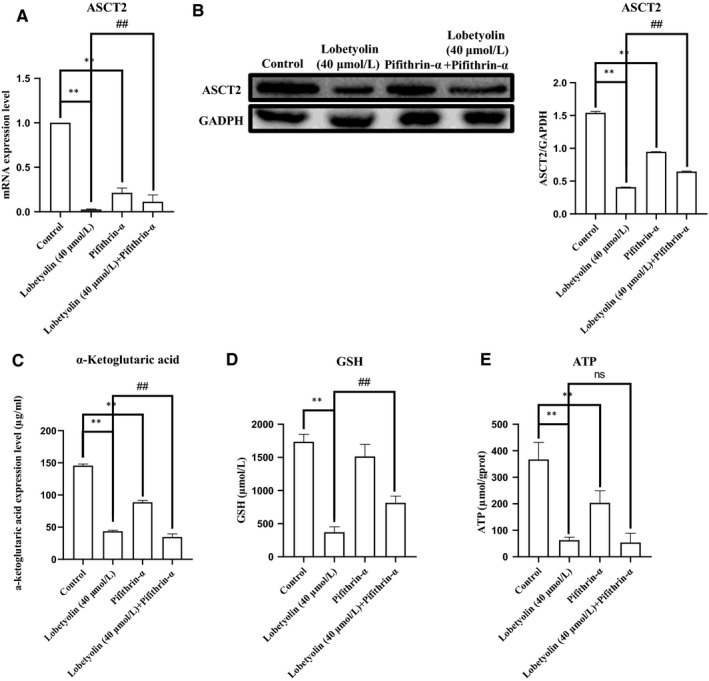
The role of p53 in Lobetyolin‐mediated apoptosis. (A) The effect of Lobetyolin, Pifithrin‐α and Lobetyolin + Pifithrin‐α on ASCT2 mRNA level in HCT‐116 cells; (B) The effect of Lobetyolin, Pifithrin‐α and Lobetyolin + Pifithrin‐α on ASCT2 protein expression levels in HCT‐116 cells by Western blot analysis; (C) The effect of Lobetyolin, Pifithrin‐α and Lobetyolin + Pifithrin‐α on the content of α‐ketoglutarate; (D) The effect of Lobetyolin, Pifithrin‐α and Lobetyolin + Pifithrin‐α on the content of GSH; (E) The effect of Lobetyolin, Pifithrin‐α and Lobetyolin + Pifithrin‐α on the content of ATP; **P < .01, compared with the control group; #*P* < .05, ##*P* < .01, compared with the Lobetyolin group

The above results indicated that Lobetyolin inhibited glutamine metabolism to induce apoptosis by suppressing ASCT2. To further explore the role of p53 during this process, Pifithrin‐α, the selective p53 inhibitor was applied. As illustrated in Figure 7A,B, the treatment with Lobetyolin (40 μmol/L), Pifithrin‐α and Lobetyolin (40 μmol/L) + Pifithrin‐α remarkably down‐regulated the ASCT2 mRNA and protein expression (*P* < .05). Of note, the ASCT2 mRNA level of Lobetyolin (40 μmol/L) + Pifithrin‐α group was obviously higher than that in Lobetyolin (40 μmol/L)‐treated cells, which suggested that p53 inhibition blocked the Lobetyolin‐mediated ASCT2 transcription and protein expression.

We further examined the levels of α‐ketoglutarate, ATP and GSH. As depicted in Figure 7C‐E, the concentration of α‐ketoglutarate was reduced (*P* < .01) by the exposure to Lobetyolin (40 μmol/L), Pifithrin‐α and Lobetyolin (40 μmol/L) + Pifithrin‐α. The incubations with Lobetyolin (40 μmol/L) and Lobetyolin (40 μmol/L) + Pifithrin‐α dramatically decreased the GSH content (*P* < .01). The challenge of Lobetyolin (40 μmol/L) and Lobetyolin (40 μmol/L) + Pifithrin‐α evidently restrained the generation of ATP compared with control group (*P* < .01). The administration of Lobetyolin (40 μmol/L) and Lobetyolin (40 μmol/L) + Pifithrin‐α also repressed the content of GSH vs control group (*P* < .01), which was more potent than Pifithrin‐α group (*P* < .01). Meanwhile, the exposure of Lobetyolin (40 μmol/L) + Pifithrin‐α group was significantly different from Lobetyolin (40 μmol/L) group at ATP and GSH concentration (*P* < .01). These data proved that the inhibition of p53 could suppress the Lobetyolin‐mediated glutamine metabolism in HCT‐116 cells.

**Figure 8:**
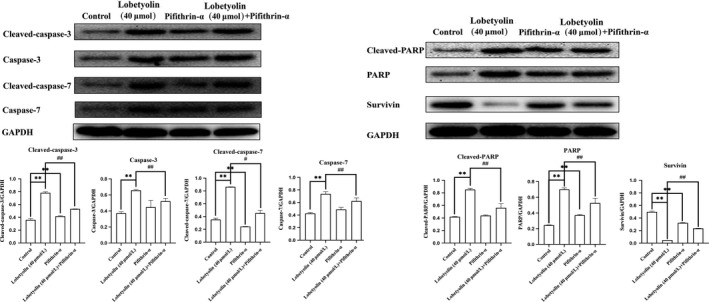
The role of p53 in Lobetyolin‐mediated apoptosis‐related protein expressions. The effect of Lobetyolin, Pifithrin‐α and Lobetyolin + Pifithrin‐α on the protein expressions of cleaved‐caspase‐3, caspase‐3, cleaved‐caspase‐7, caspase‐7, cleaved‐PARP, PARP and survivin proteins in HCT‐116 cells by Western blot analysis. **P < .01, compared with the control group; #*P* < .05, ##*P* < .01, compared with the Lobetyolin group

As depicted in Figure 8, the Western blot analysis indicated that the Lobetyolin (40 μmol/L), Pifithrin‐α and Lobetyolin (40 μmol/L) + Pifithrin‐α groups all inhibited the expressions of ASCT2 (*P* < .01). Notably, the Lobetyolin (40 μmol/L) + Pifithrin‐α treatment caused less inhibitory effect than that of Lobetyolin (40 μmol/L) treatment. The data proved that the blockade of p53 obviously influenced the Lobetyolin (40 μmol/L)‐caused ASCT2 down‐regulation.

Furthermore, the treatment with Lobetyolin (40 μmol/L), Pifithrin‐α and Lobetyolin (40 μmol/L) + Pifithrin‐α effectively up‐regulated the protein expressions of cleaved‐caspase‐3, caspase‐3, cleaved‐caspase‐7, caspase‐7, cleaved‐PARP and PARP and down‐regulated the protein expression of survivin. Interestingly, the Lobetyolin (40 μmol/L) + Pifithrin‐α blunted the promotion of cleaved‐caspase‐3, caspase‐3, caspase‐7, cleaved‐PARP, PARP, cleaved‐caspase‐7 and restored the survivin expression (all *P* < .01).

### 3.6 The effect of Lobetyolin on tumour growth in vivo

**Figure 9:**
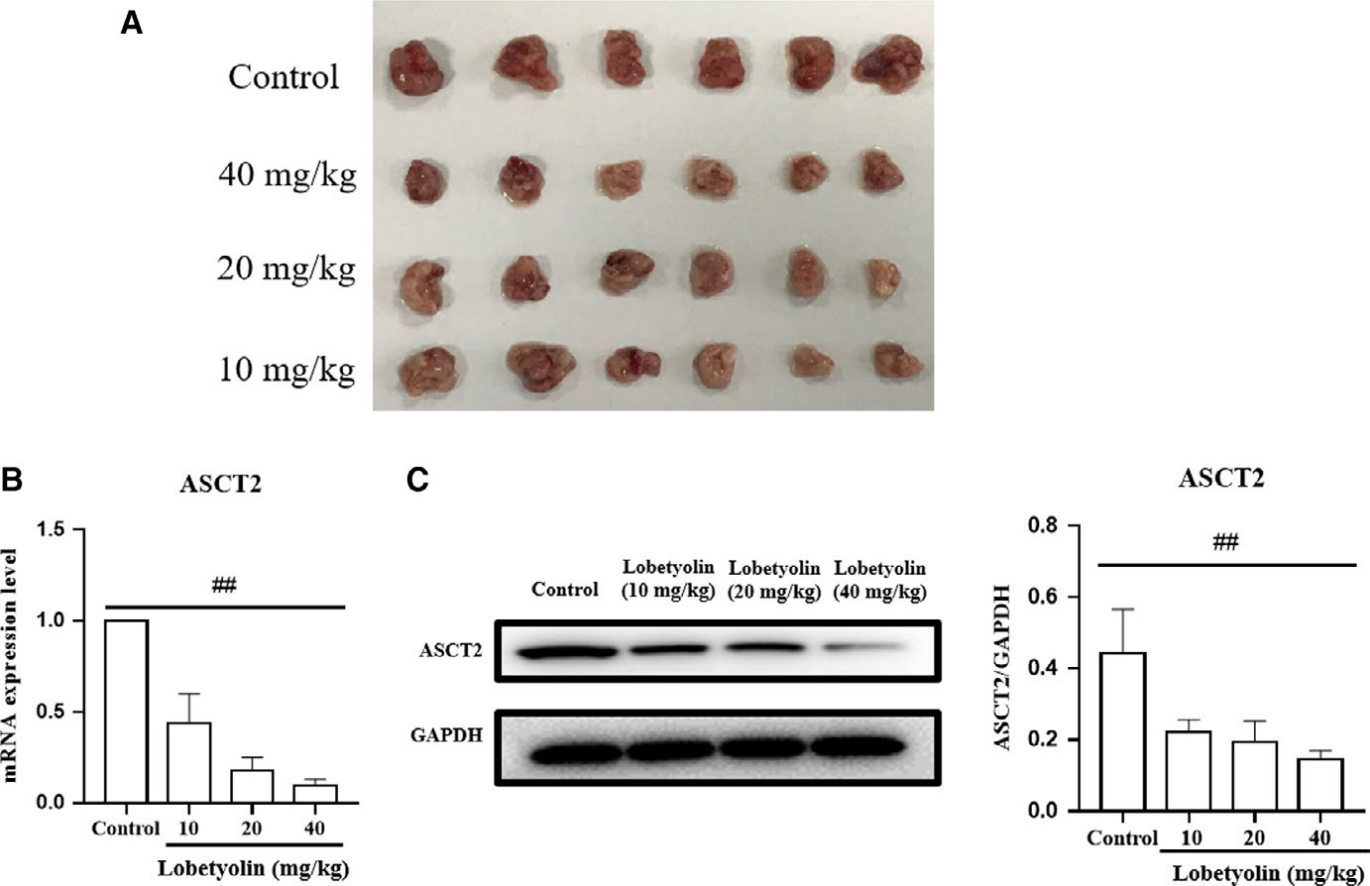
The effect of Lobetyolin on tumour growth in vivo. (A) Representative images. (B) The mRNA expression of ASCT2. (C) The protein expression of ASCT2. ##*P* < .01, compared with the control group

In order to further evaluate the antitumour effect of Lobetyolin on colon cancer in vivo, the nude (nu/nu) mice were transplanted with HCT‐116 cells. As summarized in Figure 9, the Lobetyolin (10, 20, 40 mg/kg) treatment obviously inhibited the tumour volume compared with that in control group. The ASCT2 mRNA level in Lobetyolin (10, 20, 40 mg/kg) groups was remarkably decreased compared with that in control group. Besides, the administration of Lobetyolin (10, 20, 40 mg/kg) markedly down‐regulated the ASCT2 protein expression compared with that in control group. The in vivo results suggested that Lobetyolin exerted anti‐cancer activity in nude mice, which was possibly associated with the suppression of glutamine metabolism.

## 4 DISCUSSION

Increasing evidence has emerged indicating that *C pilosula* possesses antitumour property. *Codonopsis pilosula* component inhibited the cancer cell proliferation and migration.^10^ It was proposed that *C pilosula* restrained hepatocellular carcinoma via GDF15 and HMOX1.^11^ Lobetyolin also presented cytotoxic activity against lung cancer.^12^ Although there was limited literature focused on the pharmacological effect of Lobetyolin on colon cancer, we assumed that Lobetyolin might function as a therapeutic candidate for colorectal tumour.

Glutamine, the critical precursor of nucleotides and proteins, is generally considered as an essential mediator for cellular metabolism. The deprivation of glutamine conduces to the reduction in viability. The augmented glutamic acid uptake and glutamine concentration were observed in cancer cells. Glutamine is also regarded as the substance required for the generation of GSH and α‐ketoglutarate. The elevated intracellular levels of glutamate and α‐ketoglutarate contribute to the enhanced ATP production in cells. In the present study, Glutamine, glutamic acid, α‐ketoglutarate, ATP and GSH were used to evaluate the glutamine metabolism.^13^, ^14^ The treatment with Lobetyolin down‐regulated the glutamine metabolism compared with control group. Besides, the inhibition of ASCT2 with Benser and the inhibition of p53 with Pifithrin‐α both suppressed the glutamine metabolism.

It was widely acknowledged that apoptosis is the active cellular death progression eliminating damaged cells owing to the physiological or pathological stimuli. Bcl‐2 family proteins are crucial protein driving intrinsic apoptosis. Bax, a pro‐apoptotic molecule, is the key member of Bcl‐2 family. The activation of Bax stimulates caspase‐3/7 and subsequently promotes the cleavage of PARP, which induces the apoptotic process.^15^ Cleaved‐PARP, cleaved‐caspase‐3 and cleaved‐caspase‐7 were applied for diagnosing the apoptosis in colorectal cancer.^16^ Survivin, the member belongs to the apoptosis inhibitor protein family, was reported to be highly related to caspase‐3/7 pathway and participate in the modulation of colon cancer.^17^ Our data proved that Lobetyolin evidently induced apoptosis by promoting the expressions of cleaved‐caspase‐3, caspase‐3, cleaved‐caspase‐7, caspase‐7, cleaved‐PARP and PARP. Nonetheless, the blockade of ASCT2 and p53 notably inhibited the apoptotic progression.

A variety of metabolic approaches have been developed as pivotal targets for anti‐cancer drugs. It is widely acknowledged that the metabolism of specific nutrients including glucose and glutamine are required for the energy generation of cancer cell. Previous investigators illuminated that the prevention of glutamine uptake could induce the apoptosis of glutamine addicted tumours.^18^ ASCT2 drives glutamine transport through nutrient transporters in diverse cancers.^19^ The silence of ASCT2 repressed intracellular glutamine accumulation and contributed to apoptotic cell death in human breast cancer.^20^ The experimental data revealed that the inhibition of ASCT2 by Benser successfully reduced the levels of glutamine metabolism indices including Glutamine, glutamic acid, α‐ketoglutarate, ATP and GSH. The combination of Lobetyolin and Benser slightly promoted apoptosis‐related protein expressions more than Lobetyolin‐treated alone group, which suggested that the ASCT2 was involved in the regulation of Lobetyolin‐mediated HCT‐116 cells.

p53, the key tumour suppressor gene, has been clarified to regulate cellular stress. p53 plays a critical role in mediating DNA repairing, cell cycle progression and apoptosis to prevent cancer development. It was elicited that p53 regulates energy metabolism and ATP synthesis, which further elevated glutathione (GSH) level in cancer cells.^21^ Nikkuni et al^22^ demonstrated that ASCT2 was highly related to the p53 expression in laryngeal squamous cell carcinoma. ASCT2 functioned as the significant prognostic and preventive factor in tongue cancer through p53.^23^ It was proposed that glutaminase was a p53 target gene for tumour suppression.^13^ The expressions of p21 and p53 were altered in glutamine‐deficient hepatoma cells.^24^ To explore the underlying p53 dependence of Lobetyolin‐mediated glutamine metabolism in colon cancer cells, Pifithrin‐α, the selective inhibitor of p53, was exposed to HCT‐116 cells. Our results displayed that the inactivation of p53 by Pifithrin‐α blocked the down‐regulation of glutamine metabolism‐associated biomarkers and apoptosis protein overexpressions, which indicated the critical role of p53 in the Lobetyolin‐modulated glutamine metabolism in HCT‐116 cells. Our in vivo experiment also proved that the ASCT2‐mediated glutamine metabolism was involved in the antitumour effect of Lobetyolin.

In conclusion, the present study demonstrated that Lobetyolin exhibited anti‐cancer effect on colon cancer HCT‐116 cells through the apoptosis regulated by ASCT2‐modulated glutamine metabolism, which was governed by p53. Further investigations are warranted before the clinical application of Lobetyolin.

## CONFLICT OF INTEREST

The authors confirm that there are no conflicts of interest.

## AUTHOR CONTRIBUTION

Wei He and Weiwei Tao conceived the experiments, contributed to research data and drafted the manuscript; Feng Zhang, Qian Jie, Wei Zhu and Yun He contributed to research data; Dongdong Sun and Haibo Cheng participated in the design and analysis; Jiani Tan Weixing Shen and Liu Li revised the manuscript. Ye Yang revised the manuscript and supervised the analysis.

## Data Availability

The data used to support findings of the study are available from the corresponding author upon request.
